# Recreation at School Age

**Published:** 1920-08-21

**Authors:** 


### THE PUBLIC WELL-BEING.

### Recreation at School Age.

A HOPE OF THE FUTURE.

The references made in these columns last week to the part played by the Boy Scout movement in providing healthy and happy recreation for children throughout the school age touched a problem which Mrs. E. M. Broome tried to grapple with in the course of an interesting address at the recent Birmingham Conference. It is an intricate problem, because it is bound up with such factors as the concentration of great masses of humanity in already overcrowded industrial centres, and intimately concerns questions so far apart as the urgent need of houses and the responsibilities of parents. It is something to the good that we have practically abolished the vicious half-time system; it is also something to the good that the compulsory period of education has been extended. But the health of the child still suffers from the extraneous duties which boys and girls of school age still have to perform, and it is perfectly obvious that until recreation in suitable surroundings is equally a part of the school curriculum and of the child's life out of school hours we shall continue, in spite of all that individual effort can accomplish, to produce a nation in which the C 3 variety predominates.

Mrs. Broome gives evidence from statistics gathered from schools in various localities (town, country, artisan, and slum districts) that in very many of these places only a few children can be said to have any play-time at all, and that is generally spent in the streets in the fine weather. A discreet silence is maintained as to the recreation obtained in the homes in bad weather. An instance was brought to notice recently in which a little girl of eight years was compelled to rise early and clean the house, upstairs and down, before going to school. Another child of the same age was left at home to put two younger children to bed and "clean up the house" while her parents went to a picture palace. The child was cleaning the hearth when her clothing caught fire, and she was burned to death. These are not isolated cases. Every social worker knows that they represent a common experience, with the inevitable consequences of a lowered vitality, a weakened constitution, and in some cases actual disease. Ignorance, even more than poverty, is at the root of this neglect and imposition; and it is neither an impracticable nor an unreasonable suggestion that laws should be framed providing that: Every child shall have a due amount of recreation, properly organised and supervised. No child shall be allowed to play in the streets. Every child shall be in bed at a certain time and have a certain amount of sleep.

Though we have not yet reached the age in which certificates for matrimony are a part of our State system, much can be done, where ignorance only is concerned, to give young people definite instruction in the continuation schools as to their prospective duties as parents. And there is no reason why compulsory attendance should not be demanded at systematic lectures in every town and village in the kingdom on this vital subject.

### The Ideal Play Centre.

The State must play a further part in behalf of the child. Mrs. Broome made a good point in showing that in the building of houses the recreative needs of the children never seem to be considered. In the town-planning and housing schemes of the immediate future play-rooms and play-grounds should form an integral part. Play centres have been established in some of the large towns, but we are only at the beginning of these things at present. Mrs. Broome pictures an ideal play centre in which there are rooms for a great variety of indoor occupations and games, with land for outdoor games, gardening, and Nature study, and even a large swimming-bath! These are possibilities of great interest, and one day we shall no doubt convert them into actualities. But they would require an enormous expenditure, and in the present state of the national finances they are not yet practical politics. In our view a more immediate service in the children's cause could be performed if the Board of Education were to make arrangements with the railway companies, and—to be thoroughly up to date—with the motor char-à-banc owners, to provide once a week for children attending school in the large industrial towns and districts—such as those of Lancashire and Yorkshire and the Midlands—to be conveyed for a day's outing into the country. This could be managed under the supervision of the teaching staffs, and one day a week could be set apart for the purpose. It would cost money, but far less than elaborate schemes of reconstruction, and it would do more than anything else to introduce the joy of health and raise the spark of hope in the lives of the little slum-dwellers.

